# Real-world discontinuation rate of teriflunomide and dimethyl fumarate in multiple sclerosis

**DOI:** 10.1177/20552173211022027

**Published:** 2021-06-14

**Authors:** Hilde Norborg, Trond Riise, Kjell-Morten Myhr, Nina Grytten, Stig Wergeland

**Affiliations:** Department of Clinical Medicine, University of Bergen, Bergen, Norway; Neuro-SysMed-Centre of Excellence for Experimental Therapy in Neurology, Department of Neurology, Haukeland University Hospital, Bergen, Norway; Norwegian Multiple Sclerosis Competence Centre, Haukeland University Hospital, Bergen, Norway; Department of Global Public Health and Primary Care, University of Bergen, Bergen, Norway; Department of Neurology, Haukeland University Hospital, Bergen, Norway; Neuro-SysMed -- Centre of Excellence for Experimental Therapy in Neurology, Department of Neurology, Haukeland University Hospital, Bergen, Norway; Department of Clinical Medicine, University of Bergen, Bergen, Norway; Neuro-SysMed - Centre of Excellence for Experimental Therapy in Neurology, Department of Neurology, Haukeland University Hospital, Bergen, Norway; Norwegian Multiple Sclerosis Competence Centre, Haukeland University Hospital, Bergen, Norway; Neuro-SysMed - Centre of Excellence for Experimental Therapy in Neurology, Department of Neurology, Haukeland University Hospital, Bergen, Norway; Neuro-SysMed - Centre of Excellence for Experimental Therapy in Neurology, Department of Neurology, Haukeland University Hospital, Bergen, Norway; Norwegian Multiple Sclerosis Registry and Biobank, Bergen, Norway

**Keywords:** Multiple sclerosis, real-world data, therapeutics, disease-modifying therapies, observational study, predictors of treatment outcomes, teriflunomide, dimethyl fumarate

## Abstract

**Background:**

For patients with MS, medication switches increase the risk of disease reactivation.

**Objective:**

Compare discontinuation rates due to treatment failure or side effects between teriflunomide and dimethyl fumarate, and investigate clinical variables affecting discontinuation rates.

**Methods:**

All patients who received teriflunomide or dimethyl fumarate at Haukeland University Hospital from 2013 until 2018 were identified. Clinical and demographic variables were extracted from the Norwegian MS Registry. Cause-specific Cox regression models estimated the rate of discontinuation due to treatment failure or side effects.

**Results:**

We included 354 patients treated with either dimethyl fumarate (*n* = 185) or teriflunomide (*n* = 169). We found 38% lower risk of discontinuation because of treatment failure for patients using dimethyl fumarate compared to teriflunomide (*p* < 0.05). In a treatment-naive subgroup (*n* = 183), we found a 38% reduced risk of discontinuation for any reason among patients using dimethyl fumarate (*p* < 0.05). There was no significant difference between treatment groups in discontinuation rate due to side effects, although more patients reported side effects when treated with dimethyl fumarate.

**Conclusion:**

Our findings suggests that dimethyl fumarate has a lower risk of discontinuation because of treatment failure among both treatment-experienced and treatment-naive patients.

## Introduction

For people with multiple sclerosis (MS), changing medication increases the risk of reactivating the disease, and tailoring treatment to each patient is therefore important.^1^ For more than a decade, injectable interferon-beta and glatiramer acetate were the only first-line therapies available. When teriflunomide (Aubagio®, Genzyme, Cambridge, MA, USA) and dimethyl fumarate (Tecfidera®, Biogen, Cambridge, MA, USA) became available as oral first-line disease-modifying treatments in Norway in 2013, many people with MS switched to these newer options since they offered no painful injections and a different side effects profile without the flu-like symptoms often experienced with the interferons.

The effectiveness of dimethyl fumarate and teriflunomide in terms of reducing the annualized relapse rate and the rate of disability progression has been considered similar.^2–5^ A recent study has also found that oral DMTs have a lower risk of discontinuation of treatment in comparison to injectable DMTs.^6^ Dimethyl fumarate and teriflunomide have not been compared directly, but real-world studies comparing both efficacy and discontinuation rates have been presented recently with mixed results.^7–11^ Variables associated with discontinuation have rarely been evaluated in these studies.

We aim to compare real-world discontinuation rates due to treatment failure or side effects between teriflunomide and dimethyl fumarate using data from the Norwegian Multiple Sclerosis Registry and Biobank. We also aim to investigate clinical variables affecting discontinuation rates, in order to help identify any patient subgroups that could reach a more stable treatment situation from one of the two first-line options.

## Methods and materials

This is a population-based retrospective observational cohort study of terifunomide and dimethyl fumarate in the treatment of multiple sclerosis.

### Study population

Eligible for the study were all MS patients, aged ≥18 years, diagnosed according to the 2005 or 2010 McDonald criteria^12,13^ who received a prescription for either dimethyl fumarate or teriflunomide at Haukeland University Hospital between 1 May 2013 and 1 February 2018. Only patients that had consented for recording in the Norwegian Multiple Sclerosis Registry were included. We identified patients from hospital administrative data provided by the Department of Neurology. The government funds the health care system in Norway, and treatment is therefore available to all legal residents of Norway. Data were retrieved from the Norwegian Multiple Sclerosis Registry complemented by hospital records, from 1 January 2018 until 1 July 2018.

Exclusion criteria included a diagnosis of primary progressive MS, lack of consent in the Norwegian Multiple Sclerosis Registry, migration out of Haukeland University Hospital’s catchment area, lack of follow-up data, and if the patient never initiated the prescribed therapy. If a patient was prescribed both treatments in the follow-up period, only the first treatment was included. Follow-up started at the initiation of treatment with either teriflunomide or dimethyl fumarate. Patients were censored at discontinuation or at the end of the follow-up period for drug survival analysis. If treatment was paused because of pregnancy and restarted after pregnancy and breastfeeding, we subtracted the period of discontinuation from the total time in treatment. We did not include relapses occurring during this period in the analysis, since they were not considered to indicate treatment failure for either treatment.

### Data collection and outcomes

We extracted patient data from the Norwegian Multiple Sclerosis Registry and validated the information by reviewing the medical records for each patient. We registered information on age, sex, MS phenotype, date of onset symptoms, date of diagnosis, number of clinical relapses, number of magnetic resonance imaging (MRI) lesions at treatment start, complete history of disease-modifying therapy prior to switching to teriflunomide or dimethyl fumarate, Expanded Disability Status Scale score (EDSS), dates of treatment start and discontinuation, and reason for switching treatment both to and from dimethyl fumarate or teriflunomide. We also registered clinical relapses and new T2 and/or T1-gadolinium weighted lesions and included them in the analysis if they occurred at least 3 months after the first dose of therapy. We defined clinical relapses based on the decision of the treating neurologist at the time of clinical evaluation. Progression of disability as measured by EDSS was defined as an increase by ≥1.0 points from a baseline EDSS score of ≤5.5, or a ≥0.5 point increase from a baseline EDSS score of >5.5, when not recorded within 30 days after the onset of a relapse.^14,15^ Side effects were registered based on the description by the treating neurologist, and side effects registered as occurring after the treatment period ended were not included.

The primary outcome of this study was discontinuation of treatment because of either treatment failure or side effects. Treatment failure was defined as new clinical relapses, new MRI T2 and/or T1-gadolinium weighted lesions and/or progression of EDSS score as described above. The secondary outcome for this study was discontinuation for any reason among treatment-naive patients.

### Statistical analysis

We compared baseline characteristics using the Mann–Whitney *U*-test for the continuous variables and Pearson’s chi-square or, if necessary, Fisher’s Exact test for the categorical variables. We analyzed the possible correlation between the continuous variables by using Spearman’s rho. We analyzed the correlations between each of the continuous variables and each of the dichotomous categorical variables by calculating point-biserial or biserial correlation coefficients. We set the significance level at a *p*-value of less than 0.05 for all analyses.

Time to discontinuation of treatment was evaluated using cox proportional hazards models. First, the proportionality assumption for each variable along with Schoenfeld’s global tests were performed. We also tested the models for outliers and influential cases before completing the analyses. There was one case with missing data, this case was not included in the survival analyses.

We used cause-specific Cox regression models, as opposed to a general model, to calculate the hazard ratios of discontinuation. This was done in order to avoid overestimating the parameters and to enable further exploration of how each covariate influence the discontinuation risk.^16^ In the cause-specific model, the hazard ratios for discontinuation are estimated by censoring the patients experiencing a competing event when it occurs. In this study, discontinuation because of treatment failure or side-effects were the two main competing risks, since a negligible proportion of patients terminated treatment for other reasons.

Time to discontinuation because of treatment failure or side effects were modelled by including the covariates sex, age (18–25/26–45/46–65/> 66), disease duration from first reported symptom, number of baseline T2 and T1-gadolinium weighted MRI lesions (≤ 9 vs > 9), EDSS score at baseline (≤1,5/2,0–3,5/4,0–5,5/≥ 6,0), number of prior relapses, number of prior DMT switches, type of treatment used last (treatment naïve/injectable treatments/high efficacy treatments) and treatment group (teriflunomide = 0, dimethyl fumarate = 1). Both final models were selected based on known influential variables combined with the Akaike information criterion (AIC).^17^

For the treatment-naïve subgroup, we performed a general Cox proportional hazard regression analysis with a stepwise forward method, since the number of patients included did not enable cause-specific analysis. Time to discontinuation of treatment was modelled by including the covariates sex, age (18–45 vs. > 45), disease duration from first reported symptom, EDSS (≤ 1,5 vs > 1,5), number of baseline T2 and T1-gadolinium weighted MRI lesions (≤ 9 vs > 9), number of prior relapses and treatment group (teriflunomide = 0, dimethyl fumarate = 1). The final model was selected based on known influential variables combined with the Akaike information criterion (AIC).

All statistical analyses were performed using IBM SPSS Statistics, version 24 and R studio, version 1.4.1106.

### Standard protocol approvals, registrations and patient consent

The Northern Norway Regional Committee for Medical and Health Research Ethics approved this study with reference number 2018/1024. All included patients have given written, informed consent to be part of the Norwegian Multiple Sclerosis Registry. The study conforms with the World Medical Association Declaration of Helsinki.

### Data availability

Anonymized data are available from the Norwegian Multiple Sclerosis Registry and Biobank on reasonable request from any qualified investigator upon approval from the Norwegian Regional Committee for Medical and Health Research Ethics.

## Results

**Figure 1.**
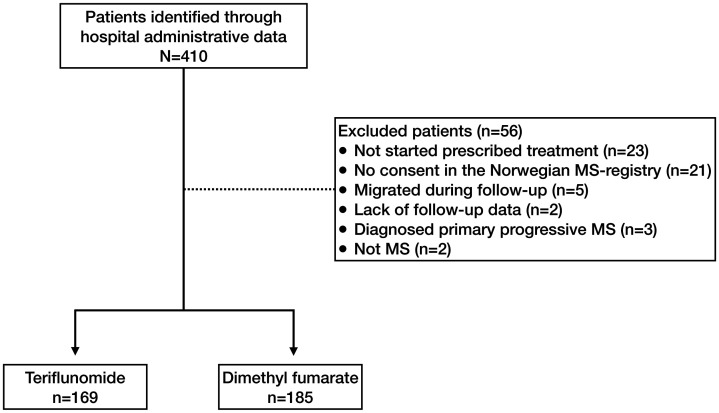
Inclusion flow chart.

We identified 410 eligible patients and excluded 56 for reasons shown in Figure 1. We therefore included 354 patients, 185 treated with dimethyl fumarate and 169 treated with teriflunomide. We excluded one patient with partly missing data in the Cox regression analyses. Patients diagnosed with radiologically isolated syndrome (n = 4) or clinically isolated syndrome (n = 10) were included. Among the included patients, eight paused therapy because of pregnancy. We followed up teriflunomide users for a mean duration of 108 (95–122) weeks and dimethyl fumarate users for 116 (104–129) weeks. The patients treated with teriflunomide were older and had a higher EDSS score when starting treatment than patients treated with dimethyl fumarate (Table 1). The same differences were also found among the treatment-naive patients. The treatment groups had different proportions of treatment-naive patients, dimethyl fumarate *n* = 85 (46%), teriflunomide *n* = 98 (58%).

**Table 1.** Baseline demographic and clinical characteristics.

		Dimethyl fumarate (n = 185)		Teriflunomide (n = 169)		
Whole cohort (N = 354)		M (SD)	n (%)	M (SD)	n (%)	*p*
Age		40.0 (12.0)		47.0 (11.0)		**<0.005**
Female sex			131 (70.8)		116 (68.6)	0.728
MS phenotype						**0.007**
	RRMS		166 (89.7)		151 (89.3)	
	SPMS		5 (2.7)		14 (8.3)	
	Other		14 (7.6)		4 (2.4)	
Disease duration (years)		8.2 (9.6)		10.0 (10.5)		0.139
Relapses		2^a^ (2)^b^		2^a^ (2)^b^		0.857
MRI lesions > 9			91 (49.2)		91 (53.8)	0.396
EDSS						**0.007**
	<1.5		114 (61.6)		73 (43.5)	
	2.0–3.5		50 (27.0)		65 (38.7)	
	4.0–5.5		11 (5.9)		18 (10.7)	
	>6		10 (5.4)		12 (7.1)	
Last DMT used						0.065
	Treatment naïve		85 (45.9)		98 (58.0)	
	Injectable		78 (42.2)		58 (34.3)	
	Other		22 (11.9)		13 (7.7)	
Number of prior DMT switches		1^a^ (2)^b^		0^a^ (1)^b^		**0.013**
Reason for last DMT switch						
	Treatment failure		29 (15.7)		16 (9.5)	0.109
	Side effects		30 (16.2)		21 (12.4)	0.364
		Dimethyl fumarate (n = 85)		Teriflunomide (n = 98)		
Treatment naïve cohort (N = 183)		M (SD)	n (%)	M (SD)	n (%)	*p*
Age		36 (12)		46 (12)	**<0.005**
Female sex			64 (75.3)		74 (72.4)	0.737
MS phenotype						0.538
	RRMS		79 (92.9)		92 (93.9)	
	SPMS		2 (2.4)		4 (4.1)	
	Other		4 (4.7)		2 (2.0)	
Disease duration (years)		3.78 (6.99)		6.85 (9.75)		**0.028**
Relapses		1^a^ (1)^b^		2^a^ (1)^b^		0.503
MRI lesions > 9			38 (44.7)		47 (48.0)	0.766
EDSS						**0.024**
	<1.5		63 (74.1)		54 (55.7)	
	2.0–3.5		17 (20.0)		38 (39.2)	
	4.0–5.5		1 (1.2)		1 (1.0)	
	>6		4 (4.7)		4 (4.1)	

Disease duration is defined as years from first symptom to treatment start. Under MS Phenotype, the “other” category includes radiologically isolated syndrome (RIS), clinically isolated syndrome (CIS) and at baseline unknown phenotype. Significant differences (p < 0.05) between dimethyl fumarate and teriflunomide patient groups are marked in bold typeface.

^a^Median.

^b^Interquartile range (IQR).

DMT: disease modifying therapy; EDSS: expanded disability status scale; M: mean; MRI: magnetic resonance imaging; MS: multiple sclerosis; RRMS: relapsing remitting multiple sclerosis; SD: standard deviation; SPMS: secondary progressive multiple sclerosis.

**Figure 2.**
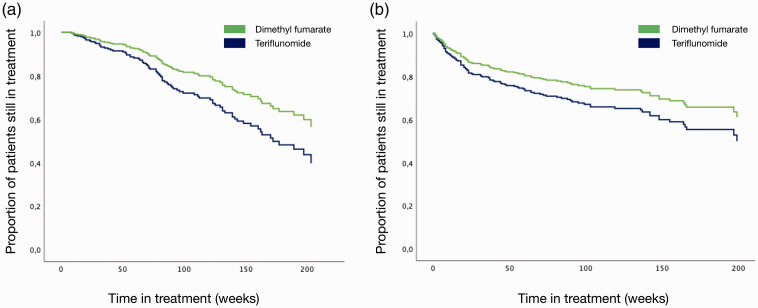
Variables affecting drug survival. Cause-specific Cox proportional hazards regression analysis showing adjusted time to discontinuation of treatment because of treatment failure (a) or side-effects (b). Both were analyzed using a stepwise-forward approach. The models were adjusted for age, sex, MS phenotype, number of previous clinical relapses, number of MRI lesions at treatment start, number of previous disease-modifying treatment switches and expanded disability status scale (EDSS) score at treatment start.

Figure 2 shows a cause-specific Cox regression analysis with time to discontinuation because of treatment failure (A) and side effects (B) adjusted for sex, age, MRI lesions at baseline, EDSS score and the number of prior relapses at baseline. Patients who received dimethyl fumarate had a significantly lower risk of discontinuation because of treatment failure than patients who received teriflunomide (adjusted HR 0.62, 95% CI 0.39–0.99) (Figure 2(a), Table 2). Patients aged 25 years and younger had a higher risk of treatment failure than older patients (*p* = 0.001, Table 2).

**Table 2.** Variables affecting drug survival.

		Crude HR (95% CI)	Adjusted HR (95% CI)	Discontinued (n)
Discontinuation due treatment failure in the whole cohort (N = 354)				
Sex	Female	(–)	(–)	56
	Male	0.99 (0.63–1.57)	0.92 (0.58–1.48)	27
Age group	18–25	(–)	(–)	11
	26–45	0.38 (0.19–0.92)*	0.33 (0.62–0.68)**	43
	46–65	0.26 (0.13–0.54)*	0.20 (0.09–0.43)**	27
	>66	0.32 (0.07–1.43)	0.19 (0.04–0.91)**	2
MRI lesions	≤9	(–)	(–)	35
	>9	1.43 (0.93–2.21)	1.58 (0.99–2.51)	48
Relapses		1.05 (0.98–1.13)	1.07 (0.99–1.15)	83
Last DMT used	Naïve	(–)	(–)	45
	Injectable	0.57 (0.36–0.92)*	0.60 (0.37–0.99)	29
	High efficacy	1.12 (0.55–2.30)	0.87 (0.37–2.02)	9
Treatment group	Teriflunomide	(–)	(–)	38
	Dimethyl fumarate	0.77 (0.50–1.19)	0.62 (0.39–0.99)*	45
Discontinuation due to side effects in the whole cohort (N = 354)				
Sex	Female	(–)	(–)	83
	Male	0.65 (0.41–1.01)*	0.59 (0.37–0.94)*	25
Age group	18–25	(–)	(–)	7
	26–45	0.93 (0.42–2.06)	0.78 (0.34–1.77)	51
	46–65	1.01 (0.45–2.23)	0.89 (0.38–2.07)	48
	>66	0.61 (0.13–2.93)	0.72 (0.14–3.64)	2
MRI lesions	≤9	(–)	(–)	56
	>9	0.93 (0.64–1.35)	0.87 (0.58–1.23)	52
Prior DMT switches		1.23 (1.08–1.41)*	1.48 (1.11–1.97)*	108
Relapses		1.05 (0.99–1.12)	0.96 (0.88–1.05)	108
Last DMT used	Naïve	(–)	(–)	50
	Injectables	0.87 (0.58–1.33)	0.54 (0.30–0.97)*	40
	High efficacy	2.16 (1.26–3.71)	0.84 (0.31–2.25)	18
Treatment group	Teriflunomide	(–)	(–)	54
	Dimethyl fumarate	0.78 (0.53–1.14)	0.71 (0.47–1.07)	54


Variables influencing the risk of treatment discontinuation because of treatment failure or side effects in the adjusted cause-specific cox proportional hazards model. (–): reference category.

Bold typeface: significant at p < 0.05 level. *: significant at p < 0.05 level; **: significant at p < 0.01 level.

CI: confidence interval; DMT: disease modifying treatment; HR: hazard ratio; MRI: magnetic resonance imaging.

There was no difference in the risk of discontinuation due to side effects between the two groups (HR 0.71, 95% CI 0.47–1.07) (Figure 2(b), Table 2). Men had a lower risk of discontinuation compared to women (HR 0.59, 95% CI 0.37–0.94). Those with previous use of any disease modifying treatment had a lower risk of discontinuation due to side effects compared to treatment naïve patients (*p* = 0.039, Table 2).

**Figure 3.**
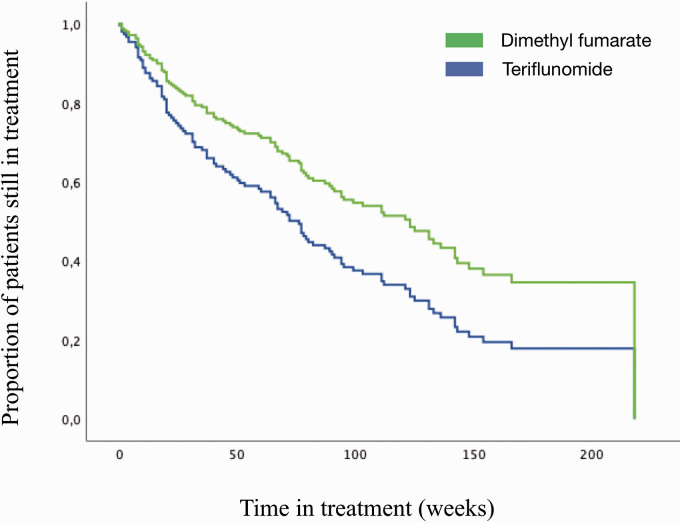
Drug survival among treatment-naive patients. Cox proportional hazards regression analysis showing adjusted time to discontinuation of treatment due to treatment failure or side effects combined among treatment naïve patients. The analysis was done using a stepwise-forward approach, adjusting for age, sex, number of previous clinical relapses, number of MRI lesions at treatment start and expanded disability status scale (EDSS) score at treatment start.

In a separate adjusted cox analysis of treatment naïve patients (dimethyl fumarate *n* = 85, teriflunomide *n* = 98), patients who received dimethyl fumarate had a significantly lower risk of discontinuation, due to treatment failure or side effects combined, than those who received teriflunomide (adjusted HR 0.62, 95% CI 0.41–0.93, *p* = 0.021), (Figure 3, Table 3). No other variables significantly affected the risk of discontinuation.

**Table 3.** Variables affecting drug survival in the treatment naïve subgroup.

Discontinuation in the treatment naïve subgroup (N = 183)		Crude HR (95% CI)	Adjusted HR (95% CI)	Discontinued (n)
Sex	Female	(–)	(–)	85
	Male	0.73 (0.46–1.15)	0.71 (0.45–1.12)	24
Age	≤45	(–)	(–)	72
	>45	0.78 (0.52–1.16)	0.66 (0.43–1.00)	37
EDSS	≤1.5	(–)	(–)	68
	>1.5	1.20 (0.81–1.78)	1.24 (0.82–1.87)	40
Relapses		1.03 (0.92–1.16)	–	109
Treatment group	Teriflunomide	(–)	(–)	57
	Dimethyl fumarate	0.67 (0.45–0.99)	0.62 (0.41–0.93)*	52


Variables influencing the risk of treatment discontinuation for any reason, including treatment failure or side effects, among treatment naïve patients in the adjusted cox proportional hazards model. (–): reference category.

Bold typeface: significant at p < 0.05 level. *: significant at p < 0.05 level. **: significant at p < 0.01 level.

CI: confidence interval; EDSS: expanded disability status scale; HR: hazard ratio.

Table 4 shows that altogether 83.2% of patients treated with dimethyl fumarate and 60.9% treated with teriflunomide reported side effects (χ^2^ = 22.071, df = 1, *p* < 0.005). Patients receiving dimethyl fumarate most frequently reported flushing (40.1%) and gastrointestinal tract problems such as nausea, diarrhea and stomach pain (26.3%). Patients receiving teriflunomide most frequently reported gastrointestinal tract problems (24.5%) and hair loss (12.0%). Two patients treated with dimethyl fumarate experienced an immediate allergic reaction, one of whom was hospitalized. One patient using teriflunomide was admitted to hospital with toxic hepatitis. No cases of progressive multifocal leukoencephalopathy were reported in either group.

**Table 4.** Clinical side effects.

	Dimethyl fumarate n = 185		Teriflunomide n = 169	
Clinical side effects	n	%	n	%
Patients experiencing side effects	154	83.2	103	60.9
Gastrointestinal tract	76	26.3	59	24.5
Flushing	116	40.1	2	1.7
Hairloss	2	0.7	29	12.0
Exanthema	19	6.6	14	5.8
Hypertension	0	0	11	4.6
Myalgia	4	1.4	8	3.3
Increasing hepatic enzymes	0	0	7	2.9
Increasing fatigue	3	1.0	6	2.5
Psychological	5	1.7	4	1.7
Headache	2	0.7	5	2.1
Other	22	7.6	30	12.4

Clinical side effects as reported by the treating physician. Count is given as number of responses pr. side effect. Percentage is given as responses within treatment group. Side effects in the gastrointestinal tract category includes nausea, diarrhea and stomach pain. The «other» category includes among others increasing rate of infection, pounding heart, changes in menstruation, dry mucous membranes.

## Discussion

Patients receiving dimethyl fumarate had a 38% lower risk of discontinuation because of treatment failure than patients using teriflunomide. There was no difference between the two treatment groups in risk of discontinuation due to side effects. A separate analysis of treatment naïve patients showed that dimethyl fumarate users had a 38% lower risk of discontinuing treatment for any reason (treatment failure or side effects) compared to teriflunomide users.

The lower risk of discontinuation because of treatment failure among dimethyl fumarate users suggests that this therapy was more effective, as shown by others.^9–11^ One study comparing dimethyl fumarate to teriflunomide reported a significantly lower annualized relapse rate during a 2-year period.^9^ In a time-to-event study, a lower risk of relapses after 38 months was found for dimethyl fumarate in comparison with teriflunomide.^18^ A cumulative incidence of discontinuation because of disease breakthrough of 22% for teriflunomide users versus 11% for dimethyl fumarate users has also recently been reported.^10^ The latter study also found that patients using dimethyl fumarate had a 23% lower hazard of relapsing. This outcome was further supported by a study reporting that the proportion of patients with at least one new T2 lesion after 2 years was lower among dimethyl fumarate users than among teriflunomide users.^11^

Even though more patients experienced side effects from dimethyl fumarate, we found no difference in discontinuation rate because of side effects. This suggests that patients experienced the side effects caused by dimethyl fumarate as less burdensome than those caused by teriflunomide. Other recent studies supports that dimethyl fumarate and teriflunomide do not differ in side effects as the reason for discontinuing treatment.^7–9^ However, previous research has also reported side effects as the main reason for switching treatment.^7,8^ We found that previous use of other disease-modifying treatments reduced the risk of discontinuing treatment because of side effects. We propose that these patients previously had experienced challenging side effects from injectable or other disease-modifying therapies, and thus better tolerated the side effects caused by the newer medications. The women in our study had a higher risk of discontinuing treatment than men. This could mean that the type of side effects frequently experienced, such as hair loss and flushing, might be more demanding for women than for men.

Among treatment naïve patients, the risk of treatment discontinuation due to treatment failure or side effects combined, was lower when receiving dimethyl fumarate than teriflunomide. We could not perform cause-specific analysis because of the relatively low number of included patients in this subgroup. This may have resulted in overestimating the parameters calculated, and the results should therefore be interpreted with caution.^16^ Nevertheless, we found the analysis to be of interest and the effect size large enough to include the results.

We did not select the patients in this study based on narrow inclusion criteria. Our results therefore reflect a real-world experience and are thus applicable to a broader population than the more rigorously selected study populations in randomized clinical trials. However, there may be some limitations, since the groups were not comparable at treatment initiation. Patients using dimethyl fumarate were younger on average and had a lower EDSS score than those receiving teriflunomide. These differences in baseline characteristics were enhanced in the treatment naïve subgroup. The choice of treatment also shifted at our department during the observation period of this study. At first, dimethyl fumarate was the preferred medication, since the pivotal clinical trials reported a slightly higher effect on annualized relapse rate.^2,5^ Later, since 2015, administrative treatment guidelines recommended teriflunomide as the first choice due to costs, except for women of reproductive age, who are recommended dimethyl fumarate and not teriflunomide because of its known potential teratogenic effects.^19,20^

The choice of statistical analysis reflects the fact that the groups were not comparable at treatment initiation. We decided to use Cox regression models since one of our objectives was to explore how different clinical and demographic variables influence the discontinuation risk. A limitation of the analysis might be that we did not correct for propensity scores. Still, there are a few communications reporting that the Cox proportional hazards regression model might be equally good for balancing treatment groups when analyzing time-to-event data.^21,22^ The statistical model is also limited because some known confounders, such as smoking and socioeconomic status, were not taken into account, since they are incompletely reported in hospital records and the Norwegian Multiple Sclerosis Registry.

Since both teriflunomide and dimethyl fumarate are considered first-line treatment options, a further study of discontinuation and efficacy among treatment-naive patients would help clinicians and patients in choosing first-line MS treatment. A new study should also include data on socioeconomic status such as education and employment, which could be a motivating factor for patients to decide to continue treatment.^19^

## Conclusion

Our findings suggest that dimethyl fumarate has a lower risk of discontinuation because of treatment failure among both treatment-experienced and treatment-naive patients. Also, easily available clinical and demographic variables influence the risk of discontinuation.
